# Letrozole for Female Infertility

**DOI:** 10.3389/fendo.2021.676133

**Published:** 2021-06-16

**Authors:** Ai-Min Yang, Na Cui, Yi-Fei Sun, Gui-Min Hao

**Affiliations:** Department of Reproductive Medicine, The Second Hospital of Hebei Medical University, Shijiazhuang, China

**Keywords:** letrozole, ovulation induction (OI), frozen-thawed embryo transfer, fertility preservation, endometrium preparation

## Abstract

Letrozole, an aromatase inhibitor that blocks estrogen synthesis by inhibiting the final step of the estrogen biosynthetic pathway, has been used in the applications of a wide range of infertility settings. It has been more than 20 years since the initial clinical trial of letrozole for ovulation induction. In light of the accumulating clinical and basic evidence, the efficacy and safety of letrozole have been identified. This mini review focuses on our current knowledge of the applications and mechanisms of letrozole for female infertility and various questions are put forward about how letrozole could be more effectively used.

## Introduction

In 1986, a new compound was tested by Ciba-Geigy (later Novartis) in an *in vivo* assay (1). This compound, CGS 20267, now known as letrozole, was a third-generation, nonsteroidal aromatase inhibitor (1). Letyrozole was approved to be effective for a wide range of breast cancer settings, which at present it’s only registered indication (2). In 1993, letrozole was initially used in animal ovulation induction (OI) (3). In 2000, the first pilot study for the clinical use of letrozole for OI indicated a high rate of ovulation in polycystic ovary syndrome (PCOS) patients (4). In 2004, a study by Legro et al. in the New England Journal of Medicine showed that letrozole was a more effective medication for OI than clomiphene in women with PCOS. This suggested that letrozole may be a better choice as a first-line medication (5). From then on, the use of letrozole in infertility treatment has been greatly popularized, and the studies about its clinical effects and mechanisms of action continued.

Letrozole is a non-steroidal, highly selective oral aromatase inhibitor (AI), which can reversibly bind to the rate-limiting enzyme P450 aromatase in estrogen biosynthesis pathway and inhibit the conversion of testosterone to estradiol and androstenedione to estrone (6). The down-regulated estrogen increases the secretion of pituitary follicle-stimulating hormone (FSH) as feedback to stimulate ovulation. Nowadays, letrozole has been extensively used to induce ovulation in anovulatory infertility patients and to augment follicles for ovulatory women. Furthermore, letrozole is used as an adjunct for intrauterine insemination (7) and *in vitro* fertilization (IVF)/intracytoplasmic sperm injection (ICSI) cycles (8). Letrozole is also used for fertility preservation in women with estrogen-sensitive cancers. Also, studies showed the effectiveness of letrozole in endometrium preparation for frozen-thawed embryo transfer (FET) (9, 10). In this mini review, we summarize the mechanism basis and clinical effects of letrozole for female infertility and we aim to provide evidence for the application of letrozole for different settings of infertility treatment.

## Pharmacology

Letrozole’s chemical structure is 4,40-[(1H-1,2,4-triazol-1-yl) methylene] bis-benzonitrile (11). It was proven to be a highly potent inhibitor of aromatase *in vitro*, *in vivo* in animals, and humans (11). Plasma kinetics of letrozole was characterized by a fast and complete absorption (t max=1h) (the mean absolute bioavailability is 99.9%), and a rather slow elimination, the plasma half-lives of letrozole (2.5 mg once daily) are 41~48 hours after oral administration (12). The extent of letrozole absorption was not influenced by the intake of food (13). The major route of elimination is metabolism by CYP450 isoenzymes into an inactive carbinol metabolite (2). The t 1/2 of letrozole can markedly increase in hepatic impairment patients and caution is required (2).

**Figure 1:**
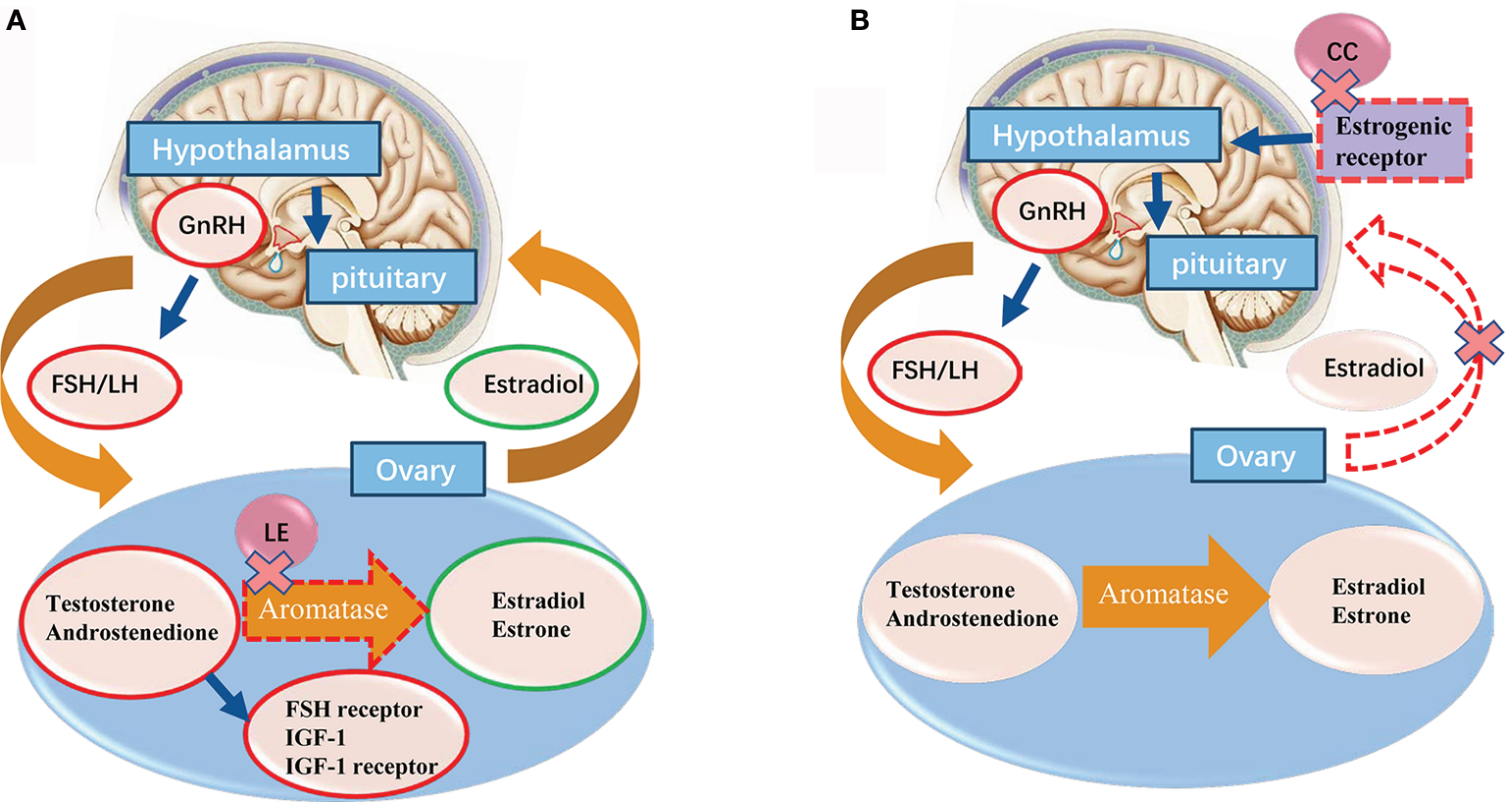
**(A)** The proposed mechanisms of letrozole for ovarian induction are centrally effect on releasing the pituitary-hypothalamic axis from estrogen negative feedback and locally effect on blocking the conversion of testosterone to estradiol and androstenedione to estrone in ovary. With the concomitant accumulation of androgens inside the ovary, promoting the follicular FSH receptor, IGF-1 and IGF-1 receptor expression, which in turn stimulates follicular growth. Normal central feedback mechanisms remain intact in letrozole ovarian induction protocol. **(B)** Clomiphene citrate administration induces gonadotropin release by binding to the estrogen receptors (ERs) in the hypothalamus, thereby blocking the negative feedback effect of estradiol. Red circle means increase, green circle means decrease. GnRH, gonadotrophin-releasing hormone; FSH, follicle-stimulating hormone; LH, lutenizing hormone; IGF, insulin-like growth factor; LE, letrozole.

Letrozole inhibits the aromatase activity by more than 99%, and endogenous estrogen synthesis by 97%-99% (12). The mechanisms of letrozole for OI remain unclear. However, it has been proposed that it may act through both centrally and peripherally mechanisms (14). Centrally, letrozole dramatically lowers the estrogen level, which prevents its negative feedback on the hypothalamic-pituitary-gonad (HPO) axis (15). Peripherally, as the conversion of androgen substrates to estrogen is inhibited, the temporary accumulation of intraovarian androgens may increase follicular sensitivity through amplification of FSH receptor gene expression (15–18). Also, androgens accumulation in the follicle may stimulate insulin-like growth factor 1 (IGF-1) and other endocrine and paracrine factors, which may synergize with FSH to promote folliculogenesis (19). The mechanism of action of letrozole for ovulation induction was shown in **Figure 1**.

Letrozole may be used alone or in conjunction with exogenous FSH for OI, but the optimal dose and regimen of letrozole is not yet clear (20). The protocol of letrozole for OI mimics the use of clomiphene citrate (CC). Typical treatment of letrozole consists of 2.5 to 7.5 mg daily taken during days 3~7 of menstrual for a 5-day course, which coincides with the availability of a 6~8mm follicle. The 6mm follicle is equipped with a high level of androgen receptors and increased androgen levels at this time promote granulosa cell mitosis and induction of FSH receptors (21–23).

## Ovlation in Induction Followed by Timed Intercourse or Intrauterine Inseimination (IUI)

CC affects the development of endometrial and cervical mucus and often leads to the development of multiple follicles (24). Besides, about 15% of PCOS patients were CC-resistant who do not respond to CC treatment (25). The mechanism of action of CC for ovulation induction was shown in **Figure 1**. Because letrozole does not inhibit negative feedback of estrogen to HPO axis, it usually induces single follicle development and avoids multiple pregnancies. On the other hand, since letrozole has a relatively short half-life (41~48 hours) (12), estrogen target tissues (such as endometrium and cervical mucus) are potentially spared adverse effects, as suggested by clinical (26) and experimental data (27). Therefore, letrozole has less effect on endometrium thickness and receptivity, and it is more conducive to embryo implantation.

The debates over the merits of letrozole and CC in anovulatory infertility have never stopped. For patients with WHO group II anovulation [WHO group anovulation classification was shown in **Table 1** (28)], there is high quality evidence that has proved that letrozole is superior to CC in terms of ovulation rate, pregnancy rate and live birth rate (29–33), but did not differ in terms of the OHSS rate (32), multiple pregnancy (32) and miscarriage rates (30–32). According to the above evidence, the current recommendation is letrozole used as first-line agent for PCOS (34) and other WHO group II anovulation patients (35).

**Table 1:** World Health Organization (WHO) group anovulation classification (28).

WHO group I	Hypogonadotrophic hypogonadism
	Idiopathic hypogonadotrophic hypogonadism Kallmann’s syndrome (isolated gonadotrophin deficiency and anosmia) Functional hypothalamic dysfunction (e. g. excessive weight loss such as in anorexia nervosa, exercise, stress, drugs, iatrogenic) Pituitary tumour, pituitary infarct (e. g. Sheehan’s syndrome)
WHO group II	Normogonadotrophic normogonadic ovarian dysfunction
	Polycystic ovary syndrome
WHO group III	Hypergonadotrophic hypogonadism (ovarian failure)
	Genetic (e. g. Turner’s syndrome) Autoimmune causes Infection (e. g. mumps oophoritis) Iatrogenic (e. g. surgical menopause, post-radiotherapy or chemotherapy) Idiopathic Other endocrinopathies, such as hyperprolactinaemia, thyroid dysfunction, other conditions of androgen excess such as congenital adrenal hyperplasia and androgen-secreting adrenal and ovarian tumours.

For ovulatory patients, letrozole is also commonly used to increase their chance of becoming pregnant. Typical diagnoses include mild male factor, endometriosis, pelvic factor, and advanced maternal age (36, 37). Despite the advantage of letrozole in PCOS patients, letrozole and CC have similar outcomes in infertile women with mild oligoasthenospermia, early-stage endometriosis, and unexplained infertility who underwent time intercourse or intrauterine insemination (IUI) (38, 39). An RCT in 2019 showed the combination of letrozole and CC was associated with a higher ovulation rate compared with letrozole alone in women with infertility and PCOS (40). However, further observation is needed on this protocol.

## Letrozole for Unexplained Infertility

The diagnosis of unexplained infertility is despite intensive evaluation of both male and female partners, the etiology may remain unknown (41). It is identified in 10%–30% of couples seeking treatment for infertility (42). A systematic review and meta-analysis in 2019 showed no difference in terms of clinical pregnancy, live birth, spontaneous miscarriage, or twin gestation between letrozole and CC for unexplained infertility (43). A Multicenter randomized controlled trial in 2020, showed that gonadotropin, clomiphene, or letrozole reached the same pregnancy outcomes (44). However, a study by Usama M Fouda et al. indicated that the extended letrozole regimen had a significantly greater pregnancy rate per cycle and cumulative pregnancy rate than CC in unexplained infertility patients underwent superovulation combined with IUI (42). But the uniform standard use of letrozole for unexplained infertility needs to be incorporated into future studies. Studies have revealed that the levels of endometrial αvβ3 expression were lower in patients with unexplained infertility than in the fertile control (45). In women with unexplained infertility treated with letrozole, the expression of αvβ3 integrin, L-selectin, leukemia inhibitory factor (LIF), and pinopod formation in epithelial and stromal was found to be significantly higher as compared to CC (46).

Letrozole would achieve mono-follicular development in most cycles, thereby may reduce multiple gestation pregnancy and OHSS, with a comparable pregnancy success rate with gonadotropins or CC, may become the first choice of treatment for unexplained infertility (41).

## Co-Administration of Letrozole During Controlled Ovarian Stimulation

Mechanistically, letrozole administration in the early follicular phase during controlled ovarian stimulation (COS) significantly increased the levels of testosterone and androstenedione in follicular fluid (47), which improved follicular sensitivity to FSH stimulation (18). For poor responders, some preliminary reports demonstrated a potential benefit of letrozole for improving ovarian response to FSH and reducing the number of gonadotropin doses but improved pregnancy outcomes (47–50).

For normal/high responders, co-treatment with letrozole significantly lower gonadotropin consumption and reduce the incidence of OHSS, the pregnancy outcomes are similar or higher than the other groups (50–52). Adjunctive use of letrozole may also be an effective means of low-cost IVF therapy particularly in ICSI cycles (8). Also, a study showed co-treatment with letrozole might revert the expression ανβ3 integrin in endometrium and improve pregnancy outcome (53). In IVM cycles, letrozole priming was not inferior to group receiving low dose FSH in pregnancy rate (54), but very few studies focus on this topic.

In 2017, a review in Cochrane included 3599 participants, found no conclusive evidence indicating that letrozole with or without gonadotropins differed from gonadotropins, either in the general population or in poor responders undergoing IVF treatment (55). And the use of letrozole may be associated with a significant increase in the incidence of cycle cancellations, as well as reductions in the mean number of oocytes retrieved (55). High-quality randomized trials are needed to reach a firm conclusion before letrozole adopted into routine clinical COS practice.

## Letrozole in the Prevention and Treatment of Ovarian Hyperstimulation Syndrome

OHSS is a potentially life-threatening complication of ovarian stimulation during the practice of assisted reproductive technologies (ART). In the rat OHSS model, letrozole decreases the level of VEGF, increases the level of PEDF (56), the combined results should lead to a decrease in the incidence of OHSS (57).

A prospective randomized controlled pilot study showed that in PCOS patients with extremely high Anti-Mullerian Hormone (AMH) levels, co-administration with letrozole results in reduced incidence of OHSS (58). However, study from Wang et al. showed that letrozole could significantly decrease the level of serum E_2_ in patients receiving freeze-all embryo strategy during luteal phase, but letrozole did not significantly reduce the rate of severe OHSS compared with the control group (59). The authors argued that, although the E_2_ level is positively correlated with the occurrence of OHSS, it is still not clear whether the high level of E_2_ is the cause or the result of OHSS. So exogenous AI therapy during the luteal phase cannot completely block OHSS in either pathogenesis or pathophysiology (59). The effectiveness and mechanisms of letrozole in the prevention and treatment of OHSS remains controversial.

## Letrozole in Preparation of Endometrium for Fet

The key to the success of FET is to improve the receptivity of the endometrium and the synchronization of endometrium and embryo development. Although Some studies showed letrozole is superior to natural or hormone-replacement therapy (HRT) in terms of clinical pregnancy, live birth (60) and miscarriage (60, 61). High-quality evidence shows no consistent advantage of any endometrial preparation has been established (62, 63). Moreover, A large meta-analysis study with a total of 31 RCTs (5426 women) in 2020 failed to show a definitive optimal protocol for endometrial preparation (64). However, letrozole is cheap, being patient-friendly, yielding at least equivalent pregnancy rates when compared with natural and artificial cycles with or without suppression, require less luteal support than artificial cycles. For anovulatory patients, it may be a better choice than HRT for FET endometrium preparation in term of patient acceptance and cost-effective analysis. More well-controlled clinical studies are needed to provide direct evidence for its advantage in endometrium preparation for FET.

## Letrozole in the Treatment of Endometriosis-Associated Infertility

AIs can suppress the locally produced E_2_ by endometriotic deposits, this makes letrozole an attractive therapy for endometriosis (65). Few studies focus on using AIs, especially letrozole, to treat endometriosis-associated infertility. In a prospective RCT, letrozole 2.5 mg/day for two months showed no difference with triptorelin and control with regard to the pregnancy rate or the disease-recurrence rate in laparoscopic and histological diagnosis of endometriosis (66). Another RCT showed superovulation with letrozole + IUI versus CC + IUI in stage I-II endometriosis demonstrated no difference in terms of pregnancy rate per cycle and cumulative pregnancy rate (37).

In summary, current evidence about using letrozole in endometriosis-associated infertility patients is limited. The efficiency of letrozole probably varies for different stages of endometriosis. More trials are warranted to provide evidence to guide our clinical management of endometriosis-associated infertility using letrozole.

## Letrozole for Fertility Preservation

With the progress of early diagnosis technology of cancer, the improvement of expected survival time, and the delay of childbearing age, the fertility preservation for young gynecological cancer patients has become an emerging need (67). In the last decades, oocyte and embryo cryopreservation have become standard procedures for fertility preservation (68). However, the standard COS regimen often stimulates the concentration of plasma estradiol and E_2_ to peak as high as 10 times of the natural cycle, which may trigger the recurrence of hormone-sensitive cancers. Compared with the standard COS regimen, letrozole combined with FSH in COS (LE-FSH-COS) significantly decreased plasma E_2_ peak concentration (69). A systematic review and meta-analysis including 2,121 hormone-sensitive cancer patients, compared the efficacy and safety of COS with letrozole *vs.* COS without letrozole, it showed the addition of letrozole did not have any negative effect on the number of mature oocytes collected and the other efficacy endpoints (70).

Recently, more and more studies demonstrated the potential beneficial use of letrozole in IVF cycles in breast cancer patients with fertility preservation treatment (69, 71, 72). Study by Oktay K et al. about fertility preservation in breast cancer patients further confirmed the effectiveness and safety of the LE-FSH-COS protocol in terms of clinical pregnancy outcomes (73). In this study, the live birth rate of the FET cycle was 45%, and there was no statistical difference between the average live birth rate (38.2%) of IVF-ET for infertile women of the same age in the United States (P =0.2). No fetal and neonatal malformation was reported either (73). However, progesterone levels were high and comparable in LE-FSH-COS protocol than conventional protocol, since progesterone has been associated with some kinds of tumor cell proliferation, caution is mandatory (71).

The effectiveness of conjunction with letrozole in patients with other hormone-sensitive cancer types has also been supported by several studies. Kawahara et al. proved that letrozole used during ovarian stimulation suppressed the growth of uterine endometrial cancer in a mouse model (74). A pilot study included six obese endometrial cancer patients who wished to preserve their fertility, treatment regimen consisted of GnRH agonist and letrozole, none of the patients had recurrences after a median follow-up of 4.0 years (range, 1.3-7.0 years), and pregnancy rate and live birth rate was 50.0% and 75.0%, respectively (75). The LE-FSH-COS regimen was used in four women with endometrial carcinoma in five IVF cycles. The protocol maintained peak E_2_ levels close to those of unstimulated cycles, at least in theory, offering a wider safety margin for endometrial cancer patients (34).

Since the effect of COS on ovarian tumors has not been determined, clinical study of LE-FSH-COS in patients with borderline ovarian tumors or invasive tumors is still lacking. Since letrozole can inhibit estrogen levels in the process of COS, theatrically letrozole may help patients with borderline ovarian tumors in reducing the risk of tumor recurrence (76). However, this hypothesis remains to be confirmed by clinical research.

## Safety

Initially, there was concern that letrozole for OI may be associated with teratogenic effects on the infants (26). However, subsequent published higher quality researches demonstrated that the rate of overall chromosomal abnormalities, congenital malformation, or adverse pregnancy and neonatal outcomes were not higher in the letrozole group than in the CC group (77–79) and the general population (80). Low-grade hot flashes, arthritis, arthralgia, and myalgia were more frequent in letrozole than placebo group in postmenopausal women with breast cancer after five years of letrozole therapy (2). Though the duration of OI using letrozole is much shorter than breast cancer treatment, study reported the sides effects are headache, hot flashes, abdominal bloating, and abdominal pain including cramps (40). Aromatase is particularly high expressed in temporal and frontal areas of the human brain, these regions are generally associated with learning, memory, sensory processing and dopaminergic activity (81). It is known that letrozole can cross the blood-brain barrier and inhibit the estrogen synthesis of hippocampal and results in cognitive dysfunction and other neurological symptoms (82–84). The study by Rune et al. showed spines, synapses, and synaptic proteins were significantly downregulated in response to letrozole and in siRNA-StAR transfected cells (85). Evidence showed a strong and significant impairment of long-term potentiation (LTP) in female mice as early as six hours after letrozole treatment, and LTP impairment was followed by loss of spine synapses in the hippocampal (86). In clinical, treatment with AI has been reported to be associated with specifically impaired hippocampus-dependent memory (87), mood disturbances, somnolence, anxiety, fatigue, and hot flashes in some studies (86, 88, 89). Though letrozole is widely used for female infertility nowadays, attention is warrant from doctors and patients about the above side effects.

Letrozole may well be teratogenically safer because its half-life virtually assures elimination from the body before implantation. But before letrozole or CC administration, pregnancy should always be ruled out (90). Further studies are needed to determine optimal dosing and long-term safety for women treated with the drug. In addition, the long-term health effects of letrozole on children need further investigation as well.

## Conclusions

As a new type of OI drug, the application of letrozole is not only limited to the clinical treatment of OI for timed intercourse but also involves many aspects of infertility treatment. Besides, letrozole is more accessible and has fewer adverse side effects and lower cost than injectable gonadotropins. Its superiority for OI in WHO group II anovulation patients has been confirmed by high-quality clinical and basic studies. The exact mechanism of OI by letrozole is not clear, the clinical applications are still in the experimental stage, and researchers have not yet reached a consensus on the standardized scheme. It is expected that large clinical samples of RCT and mechanism research will provide evidence and clear guidance for clinical application.

## Author Contributions

G-MH and A-MY conceived and designed the study. A-MY and NC wrote the manuscript. Y-FS reviewed and edited the manuscript. All authors contributed to the article and approved the submitted version.

## Funding

This study was supported by People’s Livelihood Science and Technology Project of Hebei Province (20377714D), Natural Science Foundation of Hebei Province (H2019206707 and H2019206674), National key Research and Development program (2018YFC1002104).

## Conflict of Interest

The authors declare that the research was conducted in the absence of any commercial or financial relationships that could be construed as a potential conflict of interest.
